# Protease-resistant Prion Protein in Lymphoreticular Tumors of Variant Creutzfeldt-Jakob Disease Mice

**DOI:** 10.3201/eid1203.051348

**Published:** 2006-03

**Authors:** Larisa Cervenakova, Oksana Yakovleva, Carroll McKenzie

**Affiliations:** * American Red Cross, Rockville, Maryland, USA

**Keywords:** vCJD, SJL/OlaHsd mice, PrPres, tumor, lymphoreticular tissue

## Abstract

We report protease-resistant prion protein (PrP^res^) in spontaneous lymphoreticular tumors of mice infected with the agent of variant Creutzfeldt-Jakob disease (vCJD). PrP^res^ may accumulate in lymphoreticular system tumors of asymptomatic persons with vCJD. The statistical power of estimates of vCJD prevalence might be increased by expanding screening to include samples of lymphoreticular neoplasms.

Variant Creutzfeldt-Jakob disease (vCJD) is thought to be caused by exposure to bovine products contaminated with the bovine spongiform encephalopathy agent. The prevalence of preclinical and subclinical vCJD in the United Kingdom and other European countries is still unknown. To date, all tested vCJD patients have shown an accumulation of misfolded protease-resistant protein (PrP^res^), a highly reliable indicator of infection, in lymphoreticular tissues such as spleen, tonsil, lymph nodes, and appendix (*1*). Although the time PrP^res^ starts to appear in lymphoreticular tissues of infected persons is unclear, it has been found in appendixes of 2 persons 8 months and 2 years before vCJD developed (*2*), in a lymph node and the spleen of a patient who died from a nonneurologic disorder 5 years after receiving a blood transfusion from a donor in whom vCJD subsequently developed (*3*), and in the appendixes of 3 persons from a large retrospective population study (*4*).

Lymphoreticular accumulation of infectivity and PrP^res^ occur early after scrapie infection in sheep and in various experimental animal models of transmissible spongiform encephalopathies, including mice infected with the vCJD agent (*5*). The presence of infectivity and PrP^res^ in inflamed liver, pancreas, and kidney tissues has been recently observed in transgenic and spontaneous mouse models of chronic inflammation on infection with the Rocky Mountain Laboratory strain of scrapie (*6*), and PrP^res^ has been shown in mammary glands of scrapie-infected sheep with mastitis (*7*). We report the first observation of PrP^res^ in spontaneous lymphoreticular tumors of mice with vCJD.

## The Study

Experimental studies in mice were approved by the institutional animal care and use committee of the American Red Cross Holland Laboratory. Ten inbred, 7-week-old SJL/OlaHsd (Harlan, Bicester, UK) female mice closely related to the SJL/J strain, which develops spontaneous B-cell lymphomas at >8 months of age (*8**,**9*), were intracerebrally injected under isoflurane anesthesia with 1% vCJD human brain homogenate (World Health Organization reference material) (*10*) diluted in physiologic saline, while 4 control animals received physiologic saline only. Approximately 6 months after infection, visible tumors developed in the neck areas of 5 mice, 4 with vCJD and 1 control. Two of the vCJD animals were euthanized on day 199 because of rapid tumor growth (Table). The remaining mice in the vCJD group, including 2 other animals with tumors, were later euthanized or died (range 222–386 days) without noticeable signs of neurologic disease.

**Table:** Demonstration of protease-resistant prion protein (PrP^res^) in the brain and lymphoreticular tissues of SJL/Ola mice infected with the agent of variant Creutzfeldt-Jakob disease (vCJD)

Experimental group	Postinfection interval (d)	Western blot (PrP^res^)*		
		Brain	Lymphoreticular tissue	
			Spleen	Lymph node
Mice infected with vCJD agent				
1*	199	+	+	+†
2*	199	+	+	+†
3	222	+	+	Not done
4*	318	+	+	+
5	318	+	+	Not done
6	318	+	+	Not done
7	342	+	+	Not done
8*	343	+	+	+
9	382	+	+	Not done
10	386	+	+	Not done
Control mice injected with 0.9% NaCl				
1	405	–	–	Not done
2	405	–	–	Not done
3*	321	–	–	–
4	405	–	–	Not done

*Widespread tumors of lymphoreticular tissue developed. †Mouse also had PrP^res^ in tumor involving thymus.

In the control group, the animal with tumors was euthanized on day 321, and the 3 other animals without tumors were euthanized on day 405. The autopsy of all mice, infected or not, revealed hepatomegaly and splenomegaly, with various degrees of white, nodular infiltrations of the spleen. Mice with visible tumors also had massive neoplastic nodular involvement of intestinal, mesenteric, cervical, and axillary lymph nodes and thymus. Brains and spleens were removed from all mice, and neoplastic tissues involving lymph nodes were removed from 4 infected and 1 uninfected mouse, and the thymus was removed from 2 infected mice (Table). Organs were sectioned, immediately frozen on dry ice, and stored at –80°C. PrP^res^ was extracted from brains by using high-speed centrifugation, from spleens by using methanol precipitation according to previously described methods (*5*), and from tumors with the procedure applied to the brain. Western blotting (WB) was performed by using PrP^res^-specific monoclonal antibody 6H4 (Prionics, Schlieren, Switzerland) or 6D11 as previously described (*5*).

In the vCJD group, PrP^res^ was identified in the brains and spleens of all 10 mice. In 4 mice with tumors, PrP^res^ was found in neoplastic tissues of lymph nodes and also in the neoplastic thymus of 2 of the mice (Table). The Figure shows WB analysis of PrP^res^ extracted from the brain of a vCJD patient and representative tissues of a vCJD mouse with tumors. The glycosylation pattern of PrP^res^ in mouse tissues was typical of vCJD; diglycosylated isoforms predominated over monoglycosylated and unglycosylated isoforms, with the unglycosylated isoform corresponding to a 19-kDa fragment. On the basis of WB band intensity, we observed that the concentration of PrP^res^ in neoplastic lymphoreticular tissues (lanes 3–5) was similar to that seen in the human (lane 1) and mouse (lane 2) brains. Among the control mice, PrP^res^ was not detected in the brain and spleen of any animal or in neoplastic tissues of the single affected animal (Table).

**Figure:**
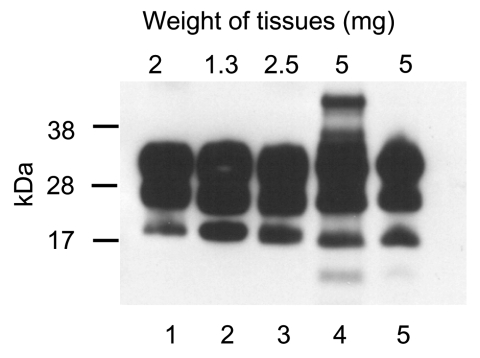
Immunoblot for protease-resistant prion protein (PrP^res^) from tissues of SJL/OlaHsd mouse infected with human variant Creutzfeld-Jakob disease (vCJD). Lanes 1–5 show representative pattern of extracted PrP^res^ after digestion with proteinase K (100 μg/mL). Lane 1, brain tissue of vCJD patient (World Health Organization reference sample). Lanes 2–5, samples from vCJD mouse in which spontaneous lymphoreticular system tumors developed: lane 2, brain; lane 3, spleen with nodular tumors; lane 4, tissue from neoplastic lymph nodes; lane 5, neoplastic thymus. The amount of original tissue used for PrP^res^ extraction is shown on the top. Samples were denatured by boiling for 10 min in Laemmli buffer containing 2% β-mercaptoethanol, resolved on NuPAGE 12% Bis-Tris gel (Invitrogen Life Technologies, Carlsbad, CA, USA), transferred to nitrocellulose membrane, and probed with anti-PrP monoclonal antibody 6D11 (dilution 1:5,000). Major glycoforms of PrP^res^ are present as 3 bands corresponding to diglycosylated, monoglycosylated, and unglycosylated molecules.

## Conclusions

Using immunohistochemical (IHC) tests, Hilton and colleagues (*1*) showed widespread PrP^res^ accumulation in the lymphoreticular system of 54 vCJD patients but not in 56 patients with familial or sporadic CJD. In contrast, when sodium phosphotungstate concentration for PrP^res^ was used to increase the sensitivity of the WB, PrP^res^ was detected in spleens of ≈30% of patients with sporadic CJD (*11*). A similar high-sensitivity detection method was used to screen 2,000 tonsils from the general population in a recently reported prospective study, with a negative result (*12*). The same method did not show PrP^res^ in the tonsils and 1 lymph node of an 83-year-old person who died from nonneurologic disease but who, 5 years before death, received a blood transfusion from a person in whom vCJD later developed (3, R. Will, pers. comm.). However, another cervical lymph node of this person tested positive for PrP^res^ by IHC test, although the appendix tested negative. This observation suggests that large retrospective and prospective studies based on screening of appendixes and tonsils with WB may not detect persons who have PrP^res^ in their lymph nodes. Estimates of prevalence of persons infected with the vCJD agent in the UK population may have been biased as a consequence of specimen selection from mostly younger participants. A retrospective study of >8,000 specimens of appendixes and tonsils included ≈70% from persons 20–29 years of age (*2*), and in a prospective study, approximately half the tonsillectomy samples came from children <9 years of age (*12*).

Our observation of the widespread presence of PrP^res^ in neoplastic lymph nodes of mice infected with the vCJD agent, and its absence in an uninfected mouse, provides experimental evidence that such tissues could be a valuable source for screening for vCJD in humans. The finding of unusually high amounts of PrP^res^ in neoplastic lymphoreticular tissues of vCJD mice, in a range comparable to that of the human and mouse brain, suggests that rapidly growing lymphoreticular tumors accumulate PrP^res^ at a high rate. Therefore, PrP^res^ might be detected in neoplastic lymphoreticular tissues of persons with vCJD. This finding is of particular importance because a recent UK study of samples collected before 1986, the years preceding the vCJD epidemic, found no PrP^res^ in lymph nodes from 58 patients with reactive conditions and 21 patients with lymphomas and carcinomas (*1*), which indicates that PrP^res^ does not spontaneously accumulate in tumors of uninfected persons. Whether PrP^res^ starts to accumulate in lymph nodes before it appears in spleens, appendixes, or tonsils of persons infected with the vCJD agent is not known. In vCJD mice, we observed PrP^res^ in the brain and neoplastic spleens and lymph nodes during at least half of the incubation period (199 days) when compared to mice with the longest survival time (>380 days). On the basis of our findings, we propose that screening of lymph node tissues from persons with reactive and neoplastic conditions and patients with various cancers with metastases in lymphoreticular organs could provide additional information, especially regarding older persons, on the prevalence of vCJD in the United Kingdom and other European countries.
